# Healthcare Waste Management: Qualitative and Quantitative Appraisal of Nurses in a Tertiary Care Hospital of India

**DOI:** 10.1155/2014/935101

**Published:** 2014-11-20

**Authors:** Siddharudha Shivalli, Vasudha Sanklapur

**Affiliations:** ^1^Community Medicine, Yenepoya Medical College, Yenepoya University, Deralakatte, Mangalore, Karnataka 575018, India; ^2^Yenepoya Medical College, Yenepoya University, Mangalore, Karnataka 575018, India

## Abstract

*Background.* The nurse's role in healthcare waste management is crucial. *Objectives.* (1) To appraise nurses quantitatively and qualitatively regarding healthcare waste management; (2) to elicit the determinants of knowledge and attitudes of healthcare waste management. *Method.* A cross-sectional study was undertaken at a tertiary care hospital of Mangalore, India. Self-administered pretested questionnaire and “nonparticipatory observation” were used for quantitative and qualitative appraisals. Percentage knowledge score was calculated based on their total knowledge score. Nurses' knowledge was categorized as excellent (>70%), good (50–70%), and poor (<50%). Chi square test was applied to judge the association of study variables with their attitudes and knowledge. *Results*. Out of 100 nurses 47 had excellent knowledge (>70% score). Most (86%) expressed the need of refresher training. No study variable displayed significant association (*P* > 0.05) with knowledge. Apt segregation practices were followed except in casualty. Patients and entourages misinterpreted the colored containers. *Conclusion.* Nurses' knowledge and healthcare waste management practices were not satisfactory. There is a need of refresher trainings at optimum intervals to ensure sustainability and further improvement. Educating patients and their entourages and display of segregation information board in local language are recommended.

## 1. Introduction

In pursuing the aim of abating health problems and enhancing the quality of care, healthcare facilities inevitably create waste that may itself be hazardous to health. Proper management of such waste is not only a legal, but also a social responsibility of the hospitals. Segregation at the site of waste generation is the first and foremost important step in healthcare waste management. It is emphasized as a means of ensuring that hazardous healthcare risk waste and healthcare general waste are separated and stored in appropriate containers. The importance of segregation is highlighted by the mere fact that only 10% to 25% of waste generated in health facilities is hazardous [1]. Failure of this vital step turns nonhazardous waste into hazardous. Segregation also enables those who handle the containers outside the hospital wards to identify and treat them appropriately. There has been a sharp increase in the amount of waste generated from both health facilities and households. It is estimated that 0.5 to 2.0 kg per bed per day hospital waste is generated in India [2].

Nursing personnel play a critical role in healthcare waste segregation in the hospitals. Their knowledge, attitudes, and practices regarding healthcare waste management are vital for the prevention of healthcare waste related hazards. Although there is an increased global awareness among health professionals about the hazards and also appropriate management techniques, the level of awareness in India is found to be below par [3–5]. Adequate knowledge about the health hazard of hospital waste, proper technique, and methods of handling the waste could go a long way toward the safe disposal of hazardous hospital waste and protect the community. With this milieu, this study was undertaken to appraise nurses with respect to healthcare waste management by both quantitative and qualitative research methods.

## 2. Objectives

The objectives of this study are as follows: (1) to appraise nurses quantitatively and qualitatively regarding healthcare waste management and (2) to elicit the determinants of their knowledge and attitudes of healthcare waste management.

## 3. Method

We conducted a hospital based cross-sectional study in a tertiary care hospital of Mangalore city in India. It is a teaching hospital attached to a medical college, recognized by the Medical Council of India, with a capacity of more than 600 beds, and generates all types of medical wastes. All the nurses (*n* = 198) working in various departments of the studied hospital formed the sampling frame. With due consideration to nurses' availability, accessibility, feasibility, and resources, we decided to include 50% (*n* = 99) of them in our study. Assuming a nonresponse rate of 15%, we approached 115 nurses of whom 100 consented to voluntary participation.

## 4. Sampling

Multistage random sampling was done. List of all the staff nurses was obtained and probability proportional to size (PPS) was applied to decide the number of nurses to be selected from each department/specialty. PPS estimated number of nurses was selected by simple random sampling in their respective department/specialty. While sampling, sex ratio among nurses was taken into account to ensure gender-wise representation. If the randomly selected nurse was not available or did not consent to voluntary participation then the next nurse in the list was included.

## 5. Study Tool

Quantitative appraisal was carried out to assess the knowledge and attitude of the nursing staff regarding healthcare waste management. A pretested self-administered questionnaire was used for this purpose. Anonymity of the study participants was maintained to enhance the participation and to ensure confidentiality. The questionnaire consisted of 18 questions of which 11 were to assess the knowledge, 5 were to assess attitudes, and 1 was to assess each of hepatitis B vaccination status and training received regarding healthcare waste management.

Knowledge assessment was done by questions pertaining to colored containers, segregation, and storage of various healthcare wastes, hazards of improper waste handling, and biohazard symbol. Attitudes were assessed by seeking their opinion regarding nurses' role in healthcare waste segregation, feeling of need for refresher training, necessity of wearing gloves, display of posters of healthcare waste segregation in hospital, and education of patients and their entourages about the same.

A scoring system was developed to assess respondents' knowledge. Each correct response was awarded with one point and zero points were given for wrong response. To score maximum (i.e., 11 points), respondents should mention four colored coded bags, that is, red, blue/white, yellow, and black (1 point); mention maximum allowed waste storage time (1 point); tell the correct colored bag to segregate used needle (1 point), paper waste (1 point), used intravenous set (1 point), discarded medicine (1 point), Foley's catheter (1 point), and dressings or cotton (1 point); know at least 3 diseases which can spread by exposure to healthcare waste (1 point); enumerate at least 3 methods of waste disposal (1 point); and identify the biohazard symbol (1 point).

## 6. Tool Validity and Reliability

Three research experts did the validation of questionnaire. Pretesting was done to verify the validity of questionnaire on ten nurses to ensure that questions were easily understood. Cronbach's alpha was computed to assess the reliability of scoring system. Obtained value of 0.763 approved the internal consistency of scoring system. Percentage score for each participant was calculated based on the total score. Study participants' knowledge was categorized as excellent, good, and poor based on their percentage scores of more than 70%, 50–70%, and less than 50%, respectively.

## 7. Statistical Analysis

Data was analyzed using Statistical Package for the Social Sciences (SPSS) for Windows, Version 16.0., SPSS Inc., Chicago. Normality test for knowledge score displayed near normal distribution. Chi square test was applied to judge the association of discrete and continuous study variables with the knowledge and attitudes of nurses, respectively. Fisher's exact test was considered when 20% or more of the cells had expected count less than 5. The level of statistical significance was set at *P* < 0.05 (two-sided).

## 8. Qualitative Appraisal

Observation is a highly valued and effective qualitative research method [6] to assess a dynamic situation like workplace practices. Qualitative appraisal was done by “nonparticipatory observation” of nursing staff in selected wards of the study hospital. Nonparticipant or direct observation is where data are collected by observing the behavior without interacting with the participants. For nonparticipatory observations, surgical and nonsurgical wards, casualty, and labour room were selected. A total of 6 wards (general surgery, general medicine, obstetrics and gynecology, pediatrics, labour room, and casualty) and injection and dressing rooms in outpatient departments were purposively chosen. For each station, “nonparticipatory observations” were done on two randomly selected nonconsecutive days of a week (i.e., 16 days total). Healthcare waste segregation practices of nurses were observed in both day and night shifts by two independent observers (first and second authors) for minimum of 2 hours every time and findings were recorded. A common checklist was used by the observers to ensure uniformity. It consisted of note on presence of 4 colored containers at site, display of segregation information board, wastes put in blue, black, red, and yellow containers, nurses' compliance with guidelines, and any other significant formative findings. At the end of every observation session, a third reviewer compared the checklist findings and both the observers discussed the findings on the same day. Consensus of findings was described in narrative form.

## 9. Results

A total of 100 nurses participated in the study and the majority (82%) of them were females. Mean age of the nurses was 25.35 ± 3.36 years and mean experience in nursing profession was 2.74 ± 2.09 years. Almost half (51%) of them were working in surgical departments and the remaining half (49%) were working in medical departments including emergency/casualty. All the nurses were given induction training regarding healthcare waste management for one day at the time of appointment by a qualified hospital administrator. As per the hospital policy all the nurses were immunized against hepatitis B.

Almost half of the nurses (47%) had excellent knowledge (>70% score) about healthcare waste management. However, one-fifth (19%) of them displayed poor knowledge (<50% score) about the same. Mean knowledge score was 69.15% (±18.6) (Table 1).

Effect of study variables such as age, sex, department of work, and nursing experience was assessed by comparing the mean knowledge scores among various categories. Knowledge was relatively better among those aged 25 years or more, female nurses, and those with the nursing experience of more than two years. However, none of the observed differences were statistically significant (*P* > 0.05) (Table 2).

**Figure 1:**
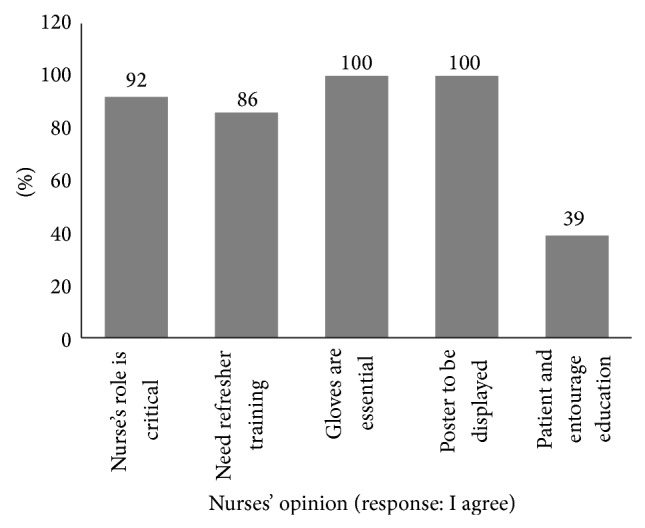
Nurses' attitudes towards healthcare waste management.

More than 90% of the nurses opined that proper healthcare waste management is imperative and could appreciate their vital role in it (Figure 1). None of them perceived “healthcare waste segregation” as a yoke in their routine work. All the study participants stressed on “use of gloves” during healthcare waste segregation and “poster display” about healthcare waste segregation in wards and hospital premises. Almost nine out of every ten nurses (86%) expressed the felt need of refresher training. Less than half of the nurses (39%) highlighted the need of patient and their entourages' education about healthcare waste management.

Nurses' attitudes were further dissected according to their age, gender, experience, specialty of work, and healthcare waste management knowledge levels (Table 3). Significantly (*P* < 0.05) higher number of nurses in surgical specialty (98%) could appreciate their vital role in healthcare waste management than their nonsurgical counterparts (85.7%). Differences observed for various attitudes within other study variables were not significant (*P* > 0.05).

Workplace practices of 31 nurses (18 surgical and 13 nonsurgical wards; 22 in day and 9 in night shifts) were appraised by “nonparticipatory observations.” They revealed the appropriateness of healthcare waste practices of nurses. In all the observed wards, nurses were using gloves while nursing the patients and handling the waste. Four colored containers (i.e., yellow, blue, black, and red) were seen and used at all the stations of observation. Instruction boards in English language were placed near the waste containers to aid nurses in proper segregation.

Wastes like dressing and cotton were placed in yellow container. Used intravenous sets and Ryle's tube and so forth contaminated with blood or body fluid were segregated in red bin. Nurses isolated waste sharps like needles in a blue puncture-proof container. However, in casualty/emergency department used sharps were placed in a cardboard box and later disposed of by shredding. No accidental needle stick injury or exposure of mucosa or skin to blood or body fluid was noted. Waste was collected from every station twice daily by the waste handlers.

Patients and their entourages are not supposed to be involved in healthcare waste segregation. However, a key formative finding was easy accessibility of colored containers to them. On more than 15 occasions ambulatory patients and their entourages put paper waste, used medicine, empty food packets, plastic waste, and so forth in wrong color bins. Many times nurses stopped them by doing so; however, on some occasions nurses could not supervise them. At the end of the observation, authors interacted with such patients or entourages and found that they were absolutely unaware of segregation and hospital waste was as good as general waste for them. Most of them could not make out the segregation display boards as they were in English.

## 10. Discussion

Responsibilities of a hospital do not end up with medical treatment only. In broader perspectives, service towards sustenance of the “good” health of the society is a default duty of any healthcare setup. In this context, proper management of biomedical wastes is of utmost public health importance [7]. The present study was an attempt to appraise nurses by mixed research methods (quantitative and qualitative) in a tertiary care hospital of Mangalore city regarding healthcare waste management. Government of India has made it a legal responsibility of healthcare facilities and set up guidelines for proper healthcare waste management [8, 9]. However, healthcare personnel's knowledge and perceived importance are crucial for its apt implementation. Findings of the study reflected unsatisfactory knowledge of the nurses. Although induction training was given for all of them, no refresher trainings were followed. The same was felt by most of the nurses (86%). It is advisable to conduct refresher trainings at optimum intervals for sustainability, improvement (19% had poor knowledge), and updating of knowledge and its implementation.

Many studies have been conducted to assess knowledge and practices of healthcare professionals. While comparability of such studies could obviously be limited (knowledge has many determinants), few may be quoted for their scope. A study by Sharma et al. [10] from Jaipur, India, among 140 dental healthcare personnel reported that for 29% of the subjects safe management of healthcare waste was not an issue at all and 36% of nurses had extremely poor knowledge about healthcare waste management. Similar findings were also reported by Bansal et al. [11] in their study from Gwalior, India. Saini et al. [12] reported positive attitudes and fair knowledge of nurses in a tertiary care set up in New Delhi. In another study on healthcare waste segregation by Deo et al. [13] nurses and lab technicians had better knowledge (90%) than medical staff like doctors (80.6%). As much as 59% of the nurses gave positive response for healthcare waste management in a study by Waseem et al. [14].

Availability and easy accessibility of all four colored containers are crucial for apt and sustained segregation practices of nurses. Similarly, the need of timely collection of segregated waste from the containers by trained waste handlers for disposal cannot be over emphasized. In this study, all the aforesaid issues were addressed to ensure sustained segregation. The cost of healthcare waste management has a significant impact on efficiency and sustainability of healthcare waste management system. A study by Maridi Eco Industries Private Limited Bangalore concluded that the cost of healthcare waste management goes down by 52.12% in common biomedical waste treatment facility (CBWTF) when compared to individual setup by a private vendor [15]. And the same is being practiced in Mangalore city.

Nurses' positive attitudes were reflected in their segregation practices. At all the observed stations (except casualty) they were compliant with segregation guidelines. However, observed practice of putting used sharps in cardboard box in casualty is not recommended. They should be immediately segregated in puncture-proof blue/white container to avoid accidental pricks. Studies conducted in Lucknow [16] and New Delhi [17] tertiary care hospitals reported use of hub cutters to mutilate the used syringes and the remaining plastic part of the syringe was treated in 1% hypochlorite solution and disposed of in red bin. In another study all the (100%) nurses were practicing according rules in a study by Saini et al. [12] from New Delhi.

Although less than half of the nurses (39%) highlighted the need of patient and their entourages' education about healthcare waste management, nonparticipation observations revealed that it is imperative. It is highly recommended to place segregation containers in such a portion of the ward where only nurses can access them. Patient and their entourages' orientation to healthcare waste and display of segregation information board in local language could further enhance the segregation efficiency and reduce the burden on nurses.

Complete hepatitis B vaccine coverage among nurses (100%) in our study is attributed to hospital policy of free immunization to high risk groups. Such policy decisions do have a positive impact on work efficiency of nurses by creating a sense of being protected. Lower level of hepatitis B vaccination among nurses was reported from studies conducted in Greece (63.25%) [18], Iran (89.6%) [19], South Africa (68%) [20], and India (44.8%) [21].

Continuous surveillance of segregation practices by hospital infection control committee and encouraging prompt reporting and also ensuring appropriate medical care for the accidental exposures among nurses could further enhance the segregation efficiency by virtue of “Hawthorne effect” (i.e., improved performance of the participant on continuous or frequent observations by the investigator) [22, 23]. A study conducted by Kumari et al. [24] underpins the same and explains the steps of establishing a dedicated biomedical waste management committee in tertiary care hospitals.

## 11. Conclusion

Nurses' knowledge and healthcare waste management practices were not satisfactory. There is a need of refresher trainings at optimum intervals to ensure sustainability and further improvement. Educating patients and their entourages and display of segregation information board in local language are recommended.

## 12. Limitation

Due to resource constraints this study was conducted in one tertiary care hospital and nonparticipatory observations were performed to appraise the segregation practices. Hence, external validity of the findings is questionable.

## Figures and Tables

**Table 1:** Knowledge level of nurses about healthcare waste management according to their percentage score.

Knowledge (% score)	Number (*N* = 100)	%
Excellent (≥70%)	47	47
Good (50–70%)	34	34
Poor (<50%)	19	19

Mean score 69.2% (±18.6)		

**Table 2:** Healthcare waste management knowledge of nurses according to their age, gender, department of work, and nursing experience.

Study variable	Number (*N* = 100)	Knowledge level						*χ* ^2^	*P*
		Poor		Good		Excellent	
		*n*	%	*n*	%	*n*	%
Age									
<25 years	52	13	25	16	30.8	23	44.2	2.562	0.278
≥25 years	48	6	12.5	18	37.5	24	50		
Gender									
Male	18	6	33.3	7	38.9	5	27.8	4.254	0.119
Female	82	13	15.9	27	32.9	42	51.2		
Department of work									
Medical	49	12	24.5	12	24.5	25	51	4.41	0.110
Surgical	51	7	13.7	22	43.1	22	43.1		
Experience in nursing									
≤2 years	61	15	24.6	20	32.8	26	42.6	3.28	0.194
>2 years	39	4	10.3	14	35.9	21	53.8		

**Table 3:** Attitudes of the nurses towards healthcare waste management according to their age, gender, department of work, nursing experience, and knowledge level^†^.

Attitudes of nurses (response: I agree)	Age (years)		Gender^§^		Specialty^¶^		Experience		Knowledge level		
	<25	≥25	M	F	Med	Surg	≤2 yrs	>2 yrs	Poor	Good	Excellent
	(*n* = 52)	(*n* = 48)	(*n* = 18)	(*n* = 82)	(*n* = 49)	(*n* = 51)	(*n* = 61)	(*n* = 39)	(*n* = 19)	(*n* = 34)	(*n* = 47)
Nurses play a critical role in healthcare waste segregation	90.4	93.8	88.9	92.7	85.7^‡^	98^‡^	91.8	92.3	89.4	94.1	94.7

Felt the need of refresher training	82.7	89.6	88.9	85.4	79.6	92.2	86.9	84.6	85.1	88.2	84.2

Wearing gloves is essential during waste segregation	98.1	100	100	98.8	98	100	98.4	100	100	100	97.9

Display of posters of healthcare waste segregation	100	100	100	100	100	100	100	100	100	100	100

Patients and entourages' education is required	38.5	39.6	44.4	37.8	34.7	43.1	39.3	38.5	36.2	47.1	31.6

^†^Expressed as percentage of each subcategory/cell; ^‡^significant (*P* < 0.05) by Fischer's exact test; ^§^M: male, F: female; ^¶^Med: medical, Surg: surgical.
